# PAR2 Activation on Human Kidney Tubular Epithelial Cells Induces Tissue Factor Synthesis, That Enhances Blood Clotting

**DOI:** 10.3389/fphys.2021.615428

**Published:** 2021-03-10

**Authors:** Abishek Iyer, Tyrone L. R. Humphries, Evan P. Owens, Kong-Nan Zhao, Paul P. Masci, David W. Johnson, David Nikolic-Paterson, Glenda C. Gobe, David P. Fairlie, David A. Vesey

**Affiliations:** ^1^Australian Research Council Centre of Excellence in Advanced Molecular Imaging, Institute for Molecular Bioscience, The University of Queensland, Brisbane, QLD, Australia; ^2^Centre for Inflammation and Disease Research, Institute for Molecular Bioscience, The University of Queensland, Brisbane, QLD, Australia; ^3^Centre for Kidney Disease Research, Translational Research Institute, Faculty of Medicine at the Princess Alexandra Hospital, The University of Queensland, Woolloongabba, QLD, Australia; ^4^Centre for Venomics Research, Faculty of Medicine, The University of Queensland, Translational Research Institute, Brisbane, QLD, Australia; ^5^Department of Nephrology, The University of Queensland at Princess Alexandra Hospital, Woolloongabba, QLD, Australia; ^6^Department of Nephrology, Monash Medical Centre and Monash University Centre for Inflammatory Diseases, Melbourne, VIC, Australia; ^7^School of Biomedical Sciences, Faculty of Medicine, The University of Queensland, Translational Research Institute, Brisbane, QLD, Australia

**Keywords:** protease, PAR2, tissue factor, kidney tubular epithelial cells, coagulation

## Abstract

Coagulation abnormalities and increased risk of atherothrombosis are common in patients with chronic kidney diseases (CKD). Mechanisms that alter renal hemostasis and lead to thrombotic events are not fully understood. Here we show that activation of protease activated receptor-2 (PAR2) on human kidney tubular epithelial cells (HTECs), induces tissue factor (TF) synthesis and secretion that enhances blood clotting. PAR-activating coagulation-associated protease (thrombin), as well as specific PAR2 activators (matriptase, trypsin, or synthetic agonist 2f-LIGRLO-NH_2_ (2F), induced TF synthesis and secretion that were potently inhibited by PAR2 antagonist, I-191. Thrombin-induced TF was also inhibited by a PAR1 antagonist, Vorapaxar. Peptide activators of PAR1, PAR3, and PAR4 failed to induce TF synthesis. Differential centrifugation of the 2F-conditoned medium sedimented the secreted TF, together with the exosome marker ALG-2 interacting protein X (ALIX), indicating that secreted TF was associated with extracellular vesicles. 2F-treated HTEC conditioned medium significantly enhanced blood clotting, which was prevented by pre-incubating this medium with an antibody for TF. In summary, activation of PAR2 on HTEC stimulates synthesis and secretion of TF that induces blood clotting, and this is attenuated by PAR2 antagonism. Thrombin-induced TF synthesis is at least partly mediated by PAR1 transactivation of PAR2. These findings reveal how underlying hemostatic imbalances might increase thrombosis risk and subsequent chronic fibrin deposition in the kidneys of patients with CKD and suggest PAR2 antagonism as a potential therapeutic strategy for intervening in CKD progression.

## Introduction

Patients with chronic kidney disease (CKD) often have blood coagulation disorders, associated with activation of the coagulation system and the local presence of activated clotting factors (Kincaid-Smith, 1972; Wendt et al., 1995; Wang et al., 1996, 1997). Fibrin deposits are frequently observed in the glomerulus, peritubular capillaries, tubular basement membrane and interstitial space in the kidney (Kincaid-Smith, 1972; Wendt et al., 1995; Wang et al., 1996, 1997; Grandaliano et al., 2000). The cellular mechanisms that leads to coagulation abnormalities, inflammation and fibrosis associated with increased risk of atherothrombosis in CKD are poorly understood.

Tissue factor (TF) is the primary physiological initiator of the injury-induced blood coagulation cascade (Grover and Mackman, 2018). It is an integral transmembrane glycoprotein, with no intrinsic protease activity, that binds and allosterically activates clotting factor VII, which in turn cleaves and activates factor X or factor IX. TF is expressed by many cell types, including in the brain, lungs, heart, kidney, and subendothelial cells of the vasculature. Under normal circumstances, cells in contact with blood do not express physiologically active TF. However, when mechanical or chemical damage of the vascular wall occurs, subendothelial TF is exposed to blood flow and binds factor VII, leading to activation of the extrinsic coagulation cascade. Low levels of TF are found in the circulation, associated predominantly with extracellular vesicles (EV) (Grover and Mackman, 2018). Further, TF positive EV are elevated in a range of conditions, including diabetes, dyslipidaemia and various malignancies (Owens and Mackman, 2011). In terms of CKD, local dysregulated TF expression in the kidneys can also increase the risk of thrombosis and lead to chronic fibrin deposition (Grover and Mackman, 2018). TF is localized to both the glomerulus and kidney tubules during various inflammatory CKDs, such as glomerular and tubulointerstitial nephritis (Lwaleed and Bass, 2006; Moussa et al., 2007). In these conditions, elevated levels of TF can also be found in the urine and are associated with phospholipid vesicles that appear to originate from cells within the kidney itself, rather than the glomerular blood filtrate (Lwaleed et al., 1999). Some studies have suggested measuring urinary TF as a clinical biomarker for glomerulonephritis (Lwaleed et al., 1997, 1998).

Coagulation proteases can modulate the function of kidney cells by activating certain cell surface receptors, including protease-activated receptors (PARs) (Vesey et al., 2007a, 2013; Eddy, 2009; Madhusudhan et al., 2016). Protease-activated receptor-2 (PAR2) is a G protein-coupled receptor abundantly expressed in the kidney (Vesey et al., 2007b; Chung et al., 2013). PAR2 can be activated by a range of serine proteases, including coagulation-associated proteases, such as clotting factor VIIa, factor Xa and matriptase (Camerer et al., 2000; Vesey et al., 2007a). Proteolytic cleavage of the N-terminal extracellular domain of PAR2 exposes a new N-terminus, which binds to the body of the receptor to trigger activation of a cascade of intracellular signaling events (Adams et al., 2011). In addition to proteases, peptides of six amino acids, such as 2-furoyl-LIGRLO-NH_2_ (2F) where O = ornithine, have been widely used as exogenous agonists to tease out roles for PAR2 in physiological and pathophysiological conditions (Barry et al., 2010; Adams et al., 2011). Adding to the complexity, PAR2 can also be activated by a thrombin-PAR1-PAR2 transactivation mechanism (Blackhart et al., 1996; O’Brien et al., 2000; Lin et al., 2013). Class A GPCRs, of which PARs are members, are known to exist in the cell membrane as homo- or hetero-dimers. When thrombin cleaves PAR1, it unmasks a tethered ligand that can, in addition to binding and activating PAR1, bind in trans to PAR2 and allosterically activate it. Within the human kidney, PAR2 is especially prominent in the proximal tubule epithelial cells of the kidney cortex and kidney vasculature and enhanced expression in these cells has been reported in various inflammatory kidney diseases (Grandaliano et al., 2003; Moussa et al., 2007; Chung et al., 2013; Oe et al., 2016; Hayashi et al., 2017; Wang et al., 2017; Watanabe et al., 2019). Although the precise functions of PAR2 in the kidney epithelium are unclear, there is evidence for PAR2 regulation of epithelial barrier function, ion channel activity, inflammation and fibrosis (Gui et al., 2003; Vesey et al., 2007a,b, 2013; Chung et al., 2013).

Here, we investigate whether PAR-activating proteases can signal locally in the kidneys to elevate TF expression and activity, and potentially enhance the risk of thrombosis. We show that PAR2 activation on HTEC induces synthesis and secretion of TF, which promotes the blood clotting cascade.

## Materials and Methods

### Materials

The PAR2 antagonists GB88 and I-191, PAR2 peptide agonist 2f-LIGRLO-NH_2_ (2F), PAR3 peptide agonist TFRGAP-NH_2_ and PAR4 peptide agonist GYPGKF-NH_2_ were synthesized, purified by reversed phase high performance liquid chromatogaphy and characterized at the Institute for Molecular Bioscience, The University of Queensland. The PAR1 activating peptide, TFLLRN-NH_2_ Cat. No. 1464/1 was obtained from Tocris Bioscience (Minneapolis, MN, United states). The recombinant human matriptase enzyme used in this study was either a generous gift from Professor John Hooper (Translational Research Institute, The University of Queensland) or purchased from R&D Systems, Cat # 3946-SEB-010 (Minneapolis, MN, United states). Vorapaxar, Cat. No. 23119 was obtained from Cayman Chemicals (MI, United states) The human TF antibody (R&D Systems, Cat #AF2339) was used at 1/2000 dilution. The ALG-2-interacting protein X (ALIX) rabbit polyclonal antibody was from Merck Millipore, (Temecula, CA, United states) and was used at 1/3000 dilution. The HRP-linked secondary antibodies were also from R&D Systems and were used at ≥1/25000 dilution. The base growth media used were Gibco DMEM/F12 (Cat # 11320) and Gibco DMEM (Cat # 11995) (ThermoFisher Scientific, Mt Waverley, Australia).

### Tubule Cell Isolation and Cell Culture

Segments of macroscopically and histologically normal kidney cortex (∼10 g) were obtained aseptically from the non-cancerous pole of adult human kidneys, removed surgically because of small kidney tumors. Patients were otherwise healthy. Informed consent was obtained prior to each operative procedure and the use of human kidney tissue for primary cell culture was approved by the Princess Alexandra Hospital Research Ethics Committee, Brisbane, Australia (ethics number: HREC/12/QPAH/125). The method for isolation and culture of human kidney tubular epithelial cells (HTEC) is described in detail elsewhere (Vesey et al., 2007b, 2013). Following isolation, cells were cultured in a serum free, hormonally defined, DMEM/F12 medium containing epidermal growth factor (20 ng/mL), insulin (5 μg/mL), transferrin (5 μg/mL), hydrocortisone (50 nM), triiodothyronine (5 pM), selenium (50 nM), penicillin (50 U/mL), and streptomycin (50 μg/mL). Cells were routinely cultured in this medium until use. At least 30 donor cell isolates were used in this study.

### Cell Treatments

All experiments were performed on confluent passage 1 or 2 HTEC cultured in 48-, 12 or 6-well plates (Corning, NY, United States). Before experimentation, cells were made quiescent by two washes followed by incubation for 24 h in basic media (DMEM medium with antibiotics). Effects of the PAR activating peptides and proteases (trypsin, matriptase, or thrombin) on TF production were measured by Western blotting and, in some cases, a TF activity assay. Analysis was at various time points post-treatment as indicated in the figure legends. The cells and cell conditioned medium were harvested according to the various techniques used and, if necessary, stored at −80°C until required for analysis. Harvested medium was centrifuged at 900 × *g* prior to storage. The inhibitors used in this study (PAR2: GB88 10 μM or I-191 10 μM; PAR1: vorapaxar 4 μM) were added 30 min prior to peptide or protease activator.

### Extracellular Vesicle Analysis

Initially to characterize the tissue factor secreted into the medium by 2F treated cells, the conditioned medium was concentrated 10-fold using either a Nanosep Centrifugal Device with a 3 kDa (OD003C34) or 100 kDa (OD100C35) molecular weight cut off membrane (Pall Melbourne Australia). If the secreted TF was 45 kDa we expected TF to pass through a 100 kDa membrane but be retained by a 3 kDa cut-off membrane. However, if secreted TF was larger than 100 kDa we expected it to be retained. For further analysis of EVs differential centrifugation was used. The conditioned medium was consecutively centrifuged at 300 × *g*, 2,000 × *g*, 20,000 × *g* (sediments microvesicles and apoptotic bodies EVs, size – 0.1–1.0 μm), and 200,000 × *g* (sediments exosomes, size – 30–100 nm).

### Quantitative Real-Time PCR (Used in the Supplementary Figure)

The method used for TF real-time quantitative PCR (qPCR) has been previously reported (Vesey et al., 2013). Total RNA was isolated using a RNeasy Mini Kit (Qiagen, Crawley United Kingdom) according to manufacturer’s instructions. RNA was reverse transcribed using Superscript III (ThermoFisher Scientific, Mt Waverley, Australia) and an oligo (dT) primer. cDNA from various cell samples were amplified by qPCR with specific primers as reported previously (Vesey et al., 2013). Relative gene expression was quantified using SYBR Green PCR master mix (Applied Biosystems, Foster City, CA, United States) on an Applied Biosystems Prism 7000 sequence detector. Amplification cycles proceeded as follows: 50°C for 2 min and 95°C for 10 min, follows by 40 cycles of 95°C for 15 s and 50°C for 1 min. cDNA levels at the linear phase of amplification were compared relative to expression of 18S or HRPT.

### Western Blot Analysis

Western blot analysis was performed on protein lysate from cells grown to confluence in six-well plates. A total of 2 mL of medium was used per well. At the end of the experiment, the medium was harvested, centrifuged at 900 × *g* and stored at −80°C. Cells were washed twice with ice-cold PBS and lysed with RIPA buffer (Sigma-Aldrich Cat # R0278), containing a protease inhibitor cocktail (Sigma-Aldrich, Cat. # P8340) and the phosphatase inhibitor NaF (10 mM) in 150 μL of lysis buffer per well. Cells were further disrupted by sonication, cell debris pelleted by centrifugation (13,000 × *g*, 20 min), and the supernatant collected. The total protein concentrations were measured using the BCA kit from (ThermoFisher Scientific, Mt Waverley, Australia). Equal amounts of concentrated, conditioned medium or cell protein (20–30 μg) were diluted in a reducing Bolt LDS sample buffer, heated to 70°C for 10 min, separated on a 4–12% NuPAGE gel (ThermoFisher Scientific, Mt Waverley, Australia) and electro-transferred to a 0.4 μm polyvinylidene difluoride membrane (Thermo Fisher Scientific, Mt Waverley, Australia). Membranes were blocked with SuperBlock^TM^ (PBST, ThermoFisher Scientific, Mt Waverley, Australia) for 1 h before being incubated with the primary antibody diluted in blocking buffer, overnight. After washing (4 × 5 min) with buffer (PBS containing 0.05% Tween-20) the appropriate secondary HRP conjugated antibody, (R&D Systems, Minneapolis, MN, United States), in blocking buffer was added to the membranes for a 40 min incubation at room temperature (RT) with gentle agitation. Membranes were washed as above before development with SuperSignal West Pico Plus, SuperSignal West Dura or SuperSignal West Femto (ThermoFisher Scientific, Mt Waverley, Australia) and CL-Xposure film (ThermFisher Mt Waverley, Australia) or Bio Rad ChemiDoc MP Imaging System.

To control for equal protein loading, some membranes were re-probed with a pan-actin monoclonal antibody (1:10000, Cat No. ACTN05C4, ThermoFisher Scientific, Freemont, CA, United States) or beta-tubulin monoclonal (1/5000, Cat No. T4026, Sigma-Aldrich, Castle Hill, NSW, Australia) diluted in blocking buffer overnight. Following washing the secondary antibody was used at a dilution of ≥ 1:25000. The actin and tubulin bands were visualized as above. For the CL-Xposure film the primary images were captured using a Canon flatbed scanner (LIDE200) and ArcSoft Photo Studio 5 software. The image size, brightness and contrast were adjusted using this software. ImageJ (NIH, United States), was used to estimate band intensity on some images. Pictures are representative images from at least three independent experiments.

### Tissue Factor Activity Assay (Factor Xa Generation Assay)

As TF has no intrinsic protease activity its measurement must be made by measuring the products of enzymes activated by TF. In this case we have measured factor Xa generation. A human TF chromogenic activity assay was adapted from the literature (Lwaleed et al., 2000). Active TF standards ranging from 7.8 to 250 pM were from Abcam (Cambridge, United Kingdom). The reaction mix consisted of 10 μL sample, 5 μL recombinant FVIIa (final concentration 10 nM; NovoSevenRT, Novo Nordisk), 10 μL of Factor X (final concentration of 100 nM, Hematologic Technologies Inc. Essex Junction VT, United States), 10 μL CaCl_2_ (final concentration of l0 mM) and 45 μL of Tris Buffered Saline. After a 1 h incubation at RT the reaction was initiated by addition of 10 μL of FXa substrate (Chromogenix S-2222). Readings at λ = 405 nM were taken at 1 min intervals, with mixing, for 40 min using a Multiskan FC Microplate reader (ThermoFisher Scientific, Freemont, CA, United States).

### Blood Clotting Assays

Blood clotting assays were used to determine whether the TF released from HTEC in response to 2F modified the coagulation process of whole blood. Blood was collected in citrated collection tubes (Greiner Bio One, Kremsmünster, Austria) without a separator by the Australian Red Cross Blood Service, blood bank. Ethical approval (HREC/08/QPAH/005) for use of human blood was granted by the Princess Alexandra Hospital Human Research Ethics Committee. In initial experiments, a simple tube mixing clotting assay was used with plain collection tubes (Greiner Bio One, Kremsmünster, Austria) pre-coated with hydrophilic surfactant (Dow Corning, Midland, MI, United States). This has been previously reported and shown to be a reliable estimate of blood clotting times (Zhao et al., 2019). To each tube, 20 μL of the 10-fold concentrated cell conditioned medium, 50 μL of 1 M CaCl_2_ and 3.95 mL of citrated normal whole blood were added. The timer was initiated on the addition of the blood. Tubes were recapped immediately after the timer was started and gently tilted every 15 s. Recalcified citrated whole blood clotting times were recorded when clotting was first observed and when a rigid clot formed. All blood clotting assays were carried out in triplicate. Thrombelastography (TEG 5000 series, Haemoscope Corporation, Niles, IL, United States) was then used to determine the efficiency and characteristics of blood coagulation. TEG^*R*^ version 4 software (TAS^TM^) was used for data analysis to capture four important parameters; R time (min), K time (min), α-angle (degrees) and maximum amplitude (MA) value (mm). The R value represents the time until the first evidence of a clot; K value is the time from the end of R until the clot reaches 20 mm, representing the speed of clot formation. The α angle is the tangent of the curve made as K is reached and MA reflects clot strength. Each reaction mix was prepared in a purpose-made disposable cup (Cat #. 6211, Haemoscope Corporation, Niles, IL, United States) with a maximum volume of 360 μL. Whole citrated blood was a component of all the assays and was kept at a constant volume of 320 μL. The order in which the other components were added was as follows; 1. Cell medium (20 μL), 2. Calcium (final concentration 20 mM), 3. Citrated whole blood (320 μL). This gives an excess of calcium over citrate. The citrated blood was inverted 5 times (10–15 s) prior to addition to the TEG cup. The addition of citrated blood starts the clotting time (R time). TEG analysis was carried out 37°C.

### Statistical Analysis

All studies were performed in at least triplicate from HTEC cultures obtained from at least three separate human donors unless otherwise indicated. Each experiment contained internal controls originating from the same culture preparation. In some cases, for the purposes of analysis, each experimental result was expressed as a change from the control value, which was regarded as 1, and analyzed independently. Results were expressed as means ± SEM. Comparisons between two groups were made using unpaired Student’s *t*-tests. GraphPad Prism version 6 was used to construct graphs and for statistical analysis. *P* values ≤0.05 were considered significant. To obtain error bars for Western blots the band density for each lane was compared to the internal control lane. The mean fold change in band density were then calculated. In most case 3 donor experiments were run in parallel on the same or parallel gels. One-way ANOVA with Dunnett’s *post hoc* test was then used to analyze the data.

## Results

### Protease Activated PAR2 Induces Synthesis of Tissue Factor in HTECs

**FIGURE 1:**
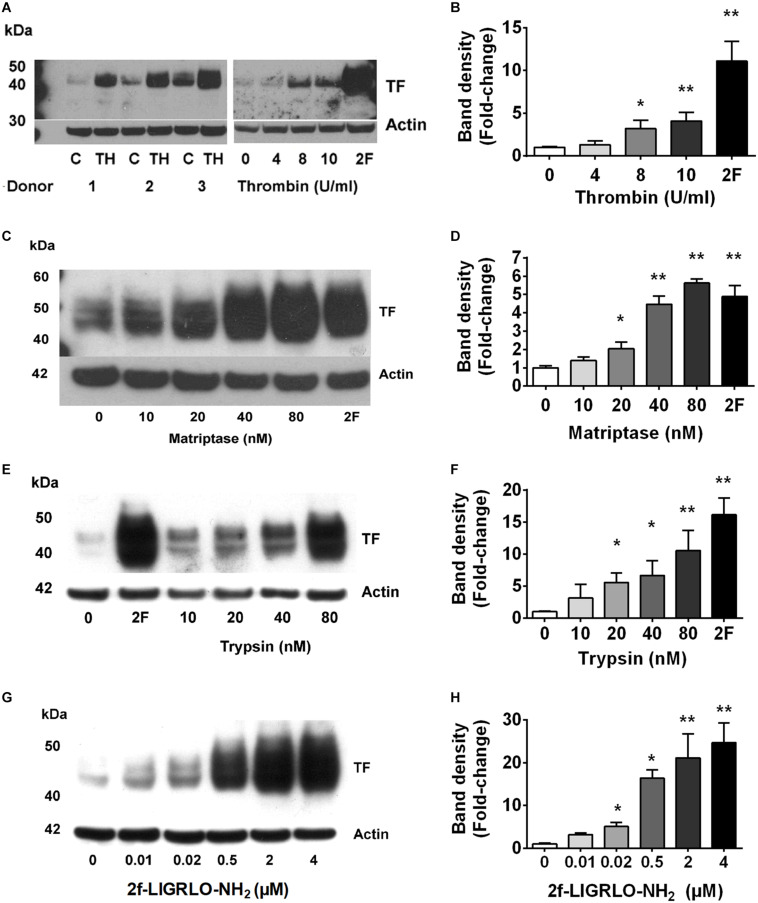
PAR2 activating proteases and peptide induces synthesis of tissue factor in HTECs. Induction of tissue factor synthesis in response thrombin **(A,B)**, matriptase **(C,D)**, trypsin **(E,F)**, and 2f-LIGRLO-NH_2_ (2F) **(G,H)** in HTEC by Western blotting. Representative blots for an individual human donor are shown for *n* ≥ 3. The graphs were established from three separate experiments and indicate changes in band density between different concentrations of the PAR2 agonists treatments. Significant (*p < 0.05 or **p < 0.01) changes in TF band density relative to actin were analyzed by one-way ANOVA with Dunnett’s *post hoc* test.

Fibrin deposits in kidneys suggest the local presence of coagulation cascade components, including TF. However, molecular mechanisms by which TF is formed, and associated coagulation abnormalities are initiated, in CKD are poorly understood. Here, we investigated whether PAR2 activation, reported to be involved in coagulation, inflammation, fibrosis and infection, could potentially promote the TF-mediated clotting cascade in the vicinity of the kidneys (Vesey et al., 2007a, 2013; Adams et al., 2011; Chung et al., 2013; Oe et al., 2016). Our previous studies and those of others have shown high levels of PAR2 expression in the kidney and HTEC (Vesey et al., 2005, 2013; Chung et al., 2013). Three PAR2-activating proteases (trypsin, matriptase, thrombin) each induced TF protein synthesis over 24 h in HTECs in a concentration-dependent manner (Figures 1A–F). Using the potent PAR2-activating synthetic peptide agonist 2F, we assessed whether specific PAR2 activation affected TF mRNA and protein synthesis in HTECs. Like the PAR2-activating proteases, the PAR2-activating peptide 2F also induced TF protein synthesis over 24 h in HTECs in a concentration-dependent manner (Figures 1G,H). Furthermore, 2F-induced an increase in TF mRNA and protein expression in HTECs in a time-dependent manner (Supplementary Figure 1). 2F induced TF mRNA expression increased 12-fold by 4 h and threefold by 24 h relative to untreated cells (Supplementary Figure 1). 2F also induced increased TF protein expression, ∼fourfold at 24 h than at 4 h. In this blot TF was not detected in control cells.

### Protease Mediated TF Synthesis Is Attenuated by PAR2 Antagonism in HTECs

**FIGURE 2:**
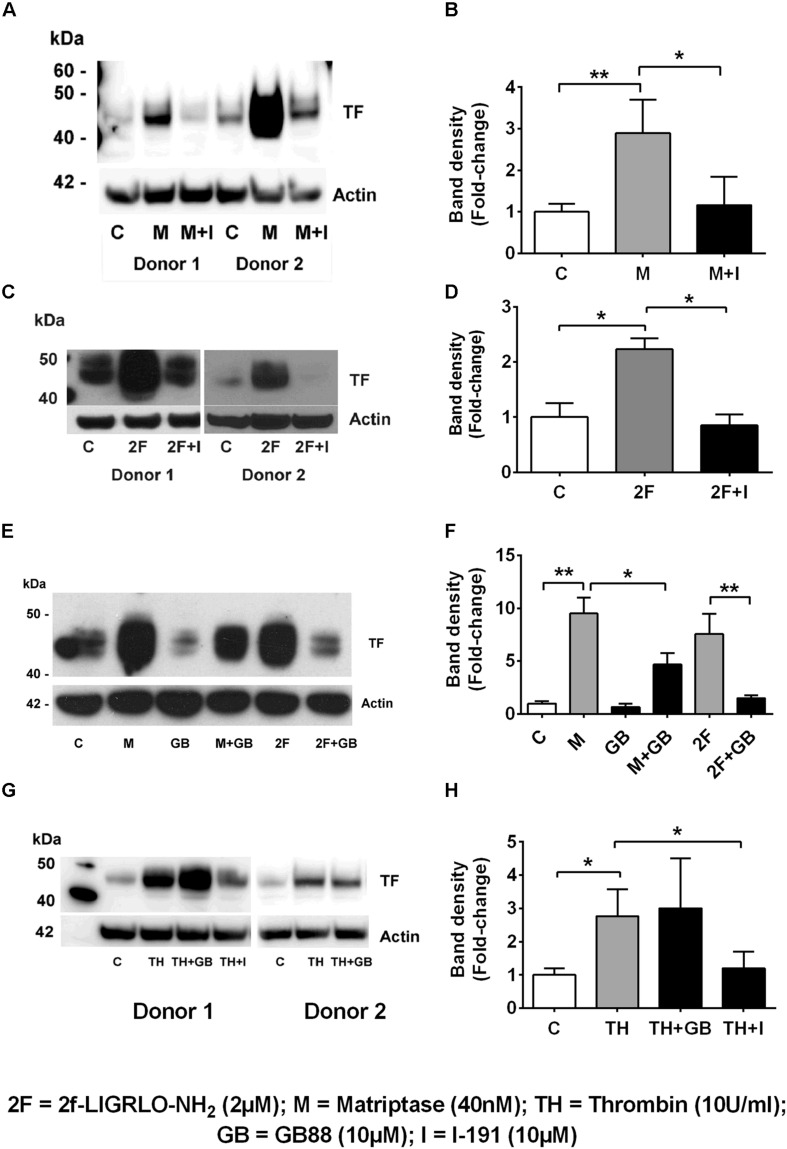
Protease-PAR2 mediated tissue factor synthesis is attenuated by PAR2 antagonists I-191 and GB88 in HTECs. **(A,B)** Matriptase (M, 40 nM) induced TF synthesis is attenuated by PAR2 antagonist I-191 (I, 10 μM). **(C,D)** 2f-LIGRLO-NH_2_, 2F induced TF synthesis is attenuated by PAR2 antagonist I-191. **(E,F)** Matriptase and 2F induced TF synthesis is attenuated by PAR2 antagonist, GB88 (GB, 10 μM). Thrombin induced TF is blocked by I-191 but not GB88 **(G,H)**. Representative blots for an individual human donor are shown, (*n* ≥ 3). Band density, fold changes were used construct graphs. * and ** indicates significant fold-changes of *p* < 0.05 and 0 < 0.01. Analysis was by an unpaired two-sided Student’s *t*-test.

We found that the soluble serine protease thrombin, the membrane bound PAR2-activating serine protease matriptase, and the potent synthetic PAR2 agonist 2F each increased expression of TF in a concentration-dependent manner in HTECs. In support of a role for PAR2 in inducing TF synthesis in HTECs, we found that two structurally unrelated PAR2 antagonists, I-191 (Jiang et al., 2018) and GB88 (Suen et al., 2014; Jiang et al., 2018) potently attenuated matriptase- and 2F-induced TF protein synthesis in HTECs (Figures 2A–F). Thrombin-induced TF synthesis was blocked by I-191, but not by GB88 (Figures 2G,H). The major TF band observed under reducing conditions on a Western blot had a molecular weight ∼45 kDa. At higher concentrations of 2F (1 μM and 4 μM), and with a longer exposure times, these bands merged to form a broad band between 40 and 60 kDa, probably reflecting variation in the extent of glycosylation. There was up to a 20-fold increase in cell-associated TF synthesis with 2F treatment compared to vehicle-treated cells (Figures 1G,H). Increased levels of cellular TF were detected within 4 h in response to as little as 10 nM 2F (Figure 1G and Supplementary Figure 1). In combination, these data reveal that thrombin, matriptase and the synthetic PAR2 agonist peptide, 2F, each induce TF protein expression in HTECs by activating PAR2.

### Thrombin Induced TF Synthesis Is Mediated by PAR1 Transactivation of PAR2

Thrombin has historically been reported as a direct activator of PAR1, 3 and 4, but not PAR2. However, recent studies have also indicated that, at least on some cells, thrombin at relatively high concentrations, can also directly activate PAR2 (Mihara et al., 2016). The transactivation of PAR2 by thrombin-activated-PAR1 has also been reported (Blackhart et al., 1996; O’Brien et al., 2000; Lin et al., 2013).

**FIGURE 3:**
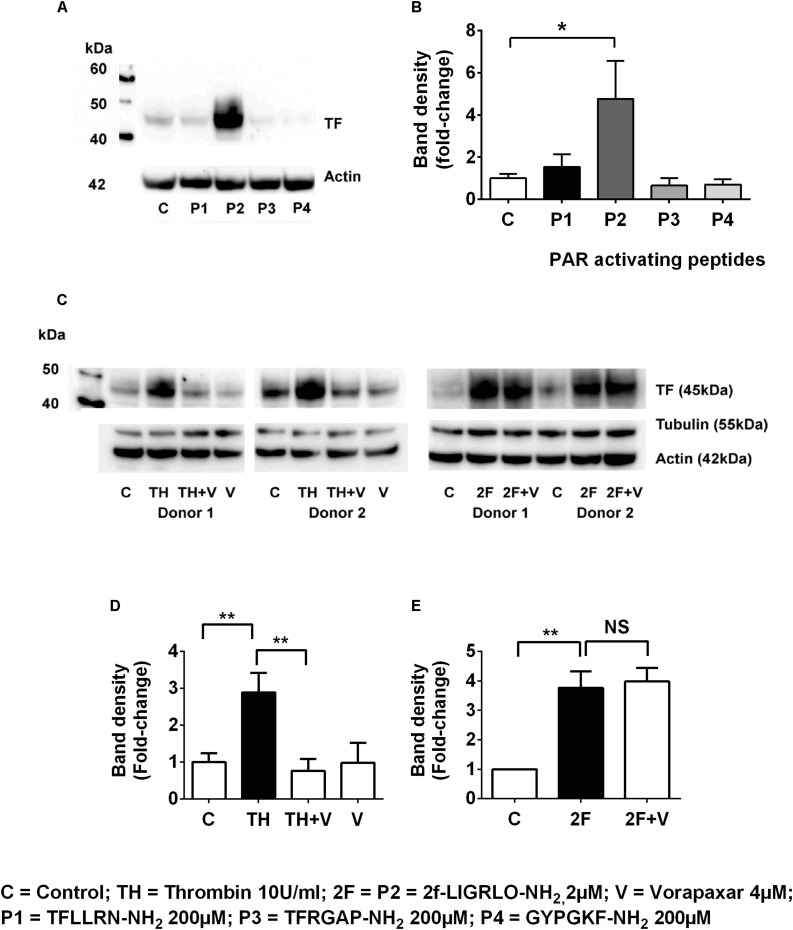
Thrombin induces tissue factor synthesis by a PAR1–PAR2 transactivation mechanism. **(A,B)** TF induction by the PAR1, PAR2 (2F), PAR3 and PAR4 activating peptides (the full sequences of these peptides are shown in the Methods section). Of the PAR-activating peptides only P2 (2F) induces TF synthesis. **(C–E)** Thrombin-induced TF synthesis is antagonized by the PAR1 antagonist Vorapaxar (4 μM) whereas 2F induced TF synthesis is not affected. Representative blots are shown (*n* ≥ 3). * and ** indicate significant fold changes of *p* < 0.05 and 0 < 0.1. An unpaired two-sided Student’s *t*-test was used.

To investigate whether other PAR members (1, 3, and 4) are capable of inducing synthesis of TF, we compared the effect on TF synthesis of an appropriate activating peptide specific for each receptor, PAR1, PAR2, PAR3, and PAR4. The PAR1-activating peptide TFLLRN-NH_2_ is reported not to cross-react with PAR2, like some other PAR-1 peptides, and unlike thrombin cannot elicit a PAR1-mediated transactivation of PAR2 (Blackhart et al., 1996). The PAR3 (TFRGAP-NH_2_) and PAR4 (GYPGKF-NH_2_) activating peptides (at 200 μM) failed to induce TF synthesis (*n* = 4, Figures 3A,B). Thus, only the peptide (2F) that activates PAR2 was capable of directly inducing TF synthesis.

In the previous section we demonstrated that thrombin induced TF was blocked by the PAR2 antagonist, I-191 (Figures 2G,H). This result led us to question whether thrombin is directly activating PAR2 or if thrombin was inducing TF by a PAR1 mediated transactivation of PAR2, We found that the PAR1 antagonist vorapaxar, was very efficient at blocking thrombin induced TF but was completely unable to effect 2F induced TF (*n* = 3, Figures 3C–E). These results are consistent with TF production by (i) a direct PAR2-activated mechanism induced by agonist 2F, and (ii) an indirect PAR2-activated mechanism induced by thrombin-activated PAR1 transactivation of PAR2 since it was inhibited by PAR1 specific antagonist vorapaxar.

### PAR2 Activation Induces Increased Secretion of Tissue Factor

**FIGURE 4:**
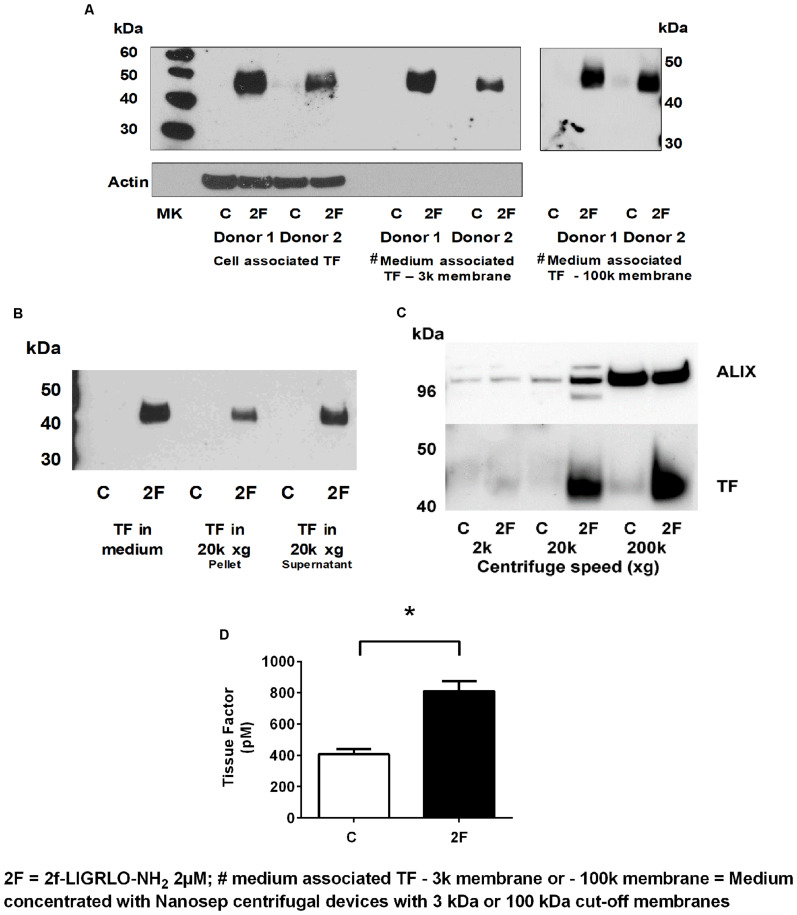
Activation of PAR2 on HTECs induces increased secretion of tissue factor. **(A–D)** 2F (2 μM) induced increased secretion of TF into cell culture medium as measured by Western blots **(A–C)**, and a tissue factor activity assay **(D)**. **(A)** Cell associated and secreted TF are the same molecular weight. TF secreted by 2F-treated cells is retained by a spin column membrane with a 3 kDa and 100 kDa cut-off membrane and in panels **(B,C)** TF is sedimented by centrifugation at 20k × *g* and 200k × *g*. Before analysis, medium was concentrated 10-fold. **(C)** The exosome marker, ALIX, is sedimented along with TF by centrifugation of conditioned medium at 200k × *g*. ALIX is equally present in both control and 2F condition medium pelleted from 200k × *g* centrifugation step, but TF is predominantly present in the 2F conditioned medium pellet. **(D)** 2F induced secretion of increased levels of active tissue factor into the cell culture medium from HTECs as measured by enzymatic production of Xa in the presence of factor VIIa. In panel **(C)** equal volumes of conditioned medium was sequentially centrifuged at 300 × *g*, 2k × *g*, 20k × *g*, and 200k × *g*. Analysis was by an unpaired two-sided Student’s *t*-test. Mean ± SEM, * indicates a significant (*p* < 0.05) difference when compared to control vehicle treatment; All the data in **(A–D)**, *n* ≥ 3. Representative blots are shown.

Since TF protein synthesis was induced in HTECs by thrombin, matriptase, trypsin, and 2F, we next investigated whether PAR2 activation on HTECs also increased secretion of TF into the culture medium. In this set of experiments, 2F was used to activate PAR2. To remove cells or cell debris that detached from the cell monolayer during the experimental period, the harvested conditioned medium was centrifuged at 900 × *g* before storage at −80°C. After concentrating the conditioned medium 10-fold, TF of the same molecular weight as cell-associated TF was detected by Western blotting (Figure 4A). This indicates that TF secretion was not initiated by proteolytic cleavage at the cell membrane. Concentrating the conditioned medium with a Nanosep centrifugal device with a 3K or 100K cut off membrane made no difference to the amount of TF detected by Western blotting suggesting that the secreted TF in conditioned medium is not present as free 45 kDa molecules, but more likely is associated with extracellular vesicles (EV, see below). If TF was secreted from cells as a free form it would be expected to pass through a membrane with a 100 kDa cut off limit membrane. This is consistent with reports that PAR2 activation induces secretion of TF positive EVs by a variety of cell lines (Al Saleh et al., 2018).

EVs are lipid bilayer-enclosed membrane vesicles ranging from 30 to 2,000 nm in diameter. They are frequently divided into three somewhat overlapping categories; apoptotic bodies (AB, 50–2,000 nm), microvesicles (MV, 50–1,000 nm) and exosomes (30–120 nm), which differ in content and mechanism of formation (Maas et al., 2017; Grover and Mackman, 2018; Hisada and Mackman, 2019). Since, TF could be secreted in association with any of these vesicles, we used differential centrifugation to identify specific EVs associated with secreted TF. Between control and 2F-treated cells there was no significant difference in the number of cells at the beginning or conclusion of experiments, or in the amount of lactate dehydrogenase released (data not shown). If the secreted TF was present in MVs, centrifugation at 20,000 × *g* would have been expected to sediment these vesicles. Indeed, TF present in conditioned medium was detected by Western blotting in the pellet derived by centrifugation at 20k × *g* (Figure 4B). Some TF was also detected in the 300 × *g* and 900 × *g* pellet associated with cells released from the monolayer (data not shown). TF secreted into conditioned medium was detectable in both the 20k × *g* pellet and its supernatant following concentration. In the 20k × *g* pellet, TF was associated predominantly with the 2F treatment, as was the protein ALG-2-interacting protein X, ALIX (see below) (Figure 4C). Finally, centrifuging the 20k × *g* supernatant at 200k × *g* led to a pellet that contained more than 50% of secreted TF (Western blotting). ALIX, which is associated with the endosomal sorting complex required for transport (ESCRT), is commonly used as a marker for exosomes. Its enrichment in the 200k × *g* pellet indicated the presence of exosomes. The amount of ALIX in the control and 2F harvested pellets was similar, suggesting that 2F does not induce enhanced exosome production but does increase the amount of TF associated with the exosomes (Figure 4C). Thus, the EVs present in the conditioned medium is possibly a mixture of AB, MV and exosomes. Using a TF activity assay, we were able to show that TF secreted from HTEC was able to bind factor VII and cause release of active factor X (Figure 4D). Taken together, this data shows that PAR2 activation induces secretion of active TF that is associated with extracellular vesicles and includes exosomes.

### PAR2 Increased Secretion of Tissue Factor Enhances Clotting of Human Whole Blood

**FIGURE 5:**
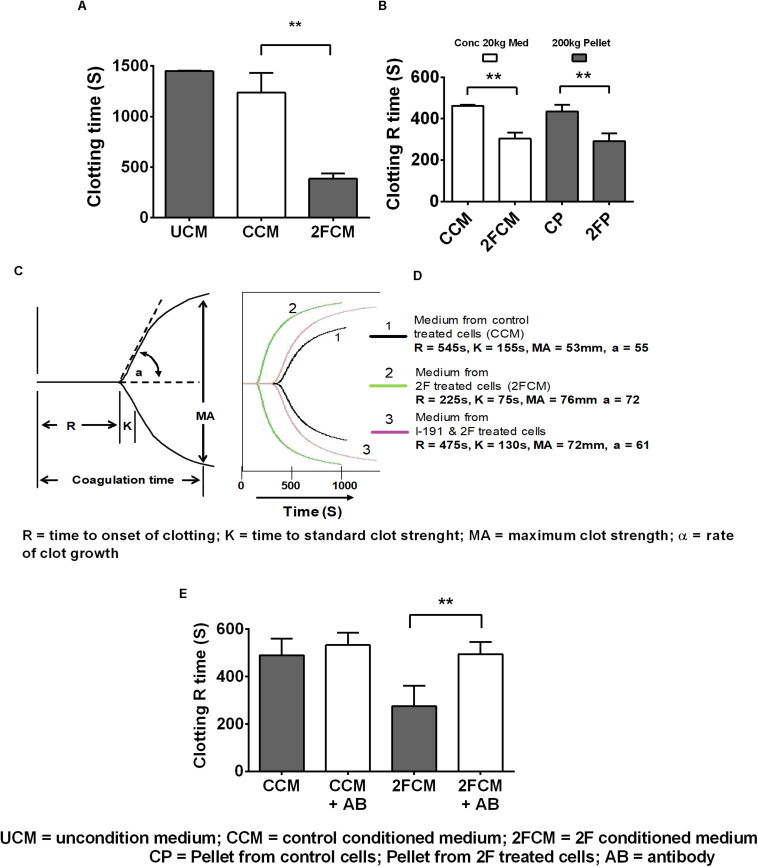
PAR2 induced increased release of tissue factor enhances clotting of whole human blood. **(A)** Clotting activity of re-calcified citrated whole-blood following addition of conditioned, concentrated (10-fold) HTEC culture medium. **(B)** TEG determination of the clotting R time of blood after addition of conditioned, concentrated medium, (following a 20k × *g* centrifugation step) or reconstituted pellets after 200k × *g* centrifugation step, *n* = 3. **(C,D)** TEG was further used to confirm the results shown in panels **(A,B)** and to further investigate the clotting parameters of blood in response to concentrated medium from cells treated with 2F, with or without addition of I-191. **(C)** diagram showing a standard TEG trace. **(D)** A typical experimental TEG trace where the green curve (2), represents medium from 2F-treated cells, showing enhanced clotting activity. Medium from cells treated with the antagonist followed by 2F – mauve curve (3) had similar clotting activity to control treated cell medium – black curve, (1). **(E)** Concentrated 2F-treated cell conditioned medium was pre-treated with or without with a TF antibody and then use in a TEG clotting assay. Data is from samples prepared from three different cell donors, i.e., *n* = 3. Blood used in panels **(A,B,D,E)** were from different donors. Ab = TF antibody. Data in panels **(A,B,E)** are presented as mean ± SEM, *n* = 3. An unpaired two-sided Student’s *t-*tests was used. Significance, *p* < 0.05, and *p* < 0.01 is indicated by * and **, respectively.

Lastly, we used whole human blood clotting assays to investigate whether 2F-induced TF secretion could induce the blood clotting cascade. In initial experiments, we used a simple timed tube clotting assay and found that conditioned media from 2F-treated cells potentiated blood clotting by threefold (1,500s to 500s) compared to vehicle treated controls (Figure 5A). This finding was confirmed using thromboelastography (TEG), which revealed similar accelerated clotting of re-calcified blood but with more detail. Interestingly, all the clotting activity present in the conditioned medium after a 20k × *g* centrifugation step could be pelleted by a subsequent centrifugation step at 200k × *g*. This is shown (Figure 5B) by a comparison of the clotting activity in the conditioned medium (concentrated after a 20k × *g* centrifugation step) with that in the reconstituted 200k × *g* centrifugation pellet. Thus, all the TF remaining in the conditioned medium after a 20k × *g* centrifugation step is associated with exosomes. A significant twofold (475 vs. 225 s) decrease in blood clotting time was observed (Figure 5C) after adding the concentrated medium sample from 2F-treated cells to whole blood in a TEG experiment. The strength of the blood clot, MA, was the same as for medium from untreated cells (>50 mm, equivalent to a full-strength clot) and there was no observable fibrinolytic activity over 3 h period, suggesting the absence of plasminogen activation. The K value (time to standard clot strength) and the α value (rate of clot growth) were both enhanced by samples from 2F-treated cells. Concentrated conditioned medium from cells pre-treated with the PAR2 antagonist I-191 before 2F addition had a clotting time like that of medium from untreated cells (Figure 5D). The modulation of the clotting time induced by concentrated medium from cells was not due to the presence of residual 2F or I-191, which had been removed by a buffer exchange step. Further, preincubating the concentrated 2F-conditioned medium with a TF-specific polyclonal antibody prevented the reduction in clotting time (Figure 5E). Taken together, these results show that PAR2 activation on HTECs induces secretion of functional TF that can initiate the clotting cascade.

## Discussion

Using primary cultures of HTEC, we have identified a potential link between PAR2 expressed on the surface of these cells with the induction of TF expression and TF stimulation of blood clotting. Induction of the clotting cascade by 2F-treated HTEC conditioned medium was attenuated by preincubation with a TF antibody or by pre-treating cells with a PAR2 antagonist. Thus, PAR2 may have a role in regulating local hemostasis by activating the TF-mediated clotting cascade in the kidney.

Our previous work revealed that HTECs in culture express functional levels of PAR1 and PAR2 at their cell surface. Both the PAR1 (TFLLRN-NH_2_) and PAR2 (2F and SLIGKV-NH_2_) activating peptides induced intracellular Ca^2+^ mobilization (iCa^2+^), but the PAR2 peptide induced larger iCa^2+^ responses than the PAR1 peptide (Vesey et al., 2005; Vesey et al., 2013). By qPCR, PAR2 is the most prominent PAR expressed by HTEC (unpublished results). Activation of PAR2 on HTEC induces iCa^2+^ and extracellular signal-regulated protein kinase (ERK1/2) mediated signaling followed by secretion of fibronectin, colony stimulating factor 2 (CSF2), C-C motif chemokine ligand 2 (CCL2), interleukin-6 (IL-6), interleukin-8 (IL-8), tumor necrosis factor-α (TNFα), and transforming growth factor-β1 (TGF-β1), suggesting a role in tubulointerstitial inflammation and fibrosis (Vesey et al., 2007b, 2013; Barry et al., 2010; Suen et al., 2012).

In normal human kidney tissue, immunohistochemistry has been used to identify TF expression in parietal and visceral epithelia of Bowman’s capsule and also in the tubular epithelium in animal models of kidney disease (Flössel et al., 1994; Wendt et al., 1995; Erlich et al., 1999). TF is constitutively expressed by tubular cell lines HKC-5, HK2 and primary kidney epithelial cells *in vitro* (Sugawara et al., 2003; Nestoridi et al., 2005; Lwaleed et al., 2007). The HTEC cultures used in the present study also constitutively expressed TF, although at relatively low levels. The constitutive expression of TF is a common feature of cells resident in highly vascularized organs, which are not normally exposed to blood (Drake et al., 1989; Parry et al., 1998; Ettelaie et al., 2016). This enables an almost instantaneous clotting event if the vasculature of an organ is compromised and provides additional hemostatic protection to that organ (Nestoridi et al., 2005; Lwaleed et al., 2007).

The proteases used in this study have been shown to activate PAR2 by cleaving the extracellular N-terminal at the common activation site, R^36^/S^37^ (Adams et al., 2011; Heuberger and Schuepbach, 2019). This unmasks a tethered ligand, SLIGKV- in humans, which binds intramolecularly and triggers a transmembrane signaling cascade mediated through G-proteins and β-arrestins (Suen et al., 2012, 2017). The specificity of these proteases for induction of TF via PAR2 was confirmed by the ability of two structurally unrelated PAR2 antagonists, I-191 and GB88, to attenuate both matriptase- and 2F-induced TF. I-191 also blocked thrombin-induced TF, whereas GB88 did not. As a biased PAR2 antagonist, GB88 has been shown to efficiently block trypsin- and 2F-induced iCa^2+^ (G_q/11_), but not activation of ERK (β-arr1/2) or Rho (G_12/13_) or cAMP (G_*o*_) induction (Suen et al., 2014). I-191 is considered a complete PAR2 antagonist as it blocks each of these signaling pathways activated by PAR2 (Jiang et al., 2018). Thrombin has historically been considered an activating protease of PAR1, PAR3 and PAR4, but not of PAR2. However, there are now numerous reports that thrombin can either directly, or indirectly, activate PAR2. Thrombin (200 nM) was reported to cleave a recombinant extracellular domain of PAR2 (Loew et al., 2000) and high concentrations of thrombin (10–50 U/ml, ∼100–500 nM) activate PAR2 (Mihara et al., 2016). Thrombin (∼10–50 nM) was reported to cleave PAR2 in a thrombomodulin dependent fashion (Heuberger et al., 2019). There are other reports that suggest that thrombin-induced responses are mediated by PAR2 (Lidington et al., 2005; Vesey et al., 2005; Sevigny et al., 2011). Thrombin is also capable of activating PAR2 by a PAR1 transactivating mechanism (O’Brien et al., 2000; Lin et al., 2013). In this scenario, thrombin induced activation of PAR1 enables the tethered ligand of PAR1 to then activate PAR2. For this mechanism to be functional, PAR1 and PAR2 must be in close proximity, likely as a heterodimer. As the PAR2 antagonist, I-191 was able to block thrombin induced TF we questioned whether thrombin directly activated PAR2 to induce TF or alternatively activated PAR2 via a PAR1 transactivating mechanism. Of the four PAR-activating peptides tested, only the PAR2-activating peptide was found to induce TF. The PAR1 antagonist Vorapaxar was able to block thrombin-induced TF synthesis, whilst not affecting 2F induced TF. These results are consistent with thrombin-induced PAR1 transactivation of PAR2 here.

Analysis of the TF present in the 2F conditioned medium revealed that it was of the same molecular weight as cell-associated TF, ∼45 kDa. This suggests that its secretion from HTEC does not involve cleavage. As a Nanosep centrifugal device with a molecular weight cut-off membrane of 100 kDa retains secreted TF, TF must be associated something that prevents if passing through the membrane pores. In this case TF was shown to be associated with EVs. By using differential centrifugation, it was found that TF could be sedimented from 2F treated HTEC conditioned medium along with EV marker protein, ALIX, confirming that TF released from HTEC is associated with EVs. Although the exact roles of EVs in physiological or pathological processes are not well defined yet, they are often described as having key roles in paracellular communication (Oggero et al., 2019). Neighboring cells and cells in distant tissues can be exposed the EVs where they can be taken up by membrane fusion or endocytosis. The fact that TF is secreted in association with EVs is likely to enhance the thrombogenic potential of TF, as it could be rapidly incorporated into the membrane of target cells. How tubular epithelial cell-derived EV could be taken up by neighboring cells, including vascular cells, and influence thrombosis is unclear. However, they might increase exposure of TF to the circulation. The enhanced permeability of the vascular endothelium in injury or disease may further increase exposure to the TF via EVs. A number of other coagulation proteases, such as FVII, FX and FII, are needed in order for TF to induce a coagulation cascade and ultimately induce fibrin deposition. It is possible that circulating clotting factors could gain accesses to the kidney interstitium in acute kidney injury or other inflammatory conditions when the kidney vasculature is compromised. The fact that TF is induced in these cells by PAR2 activation has the potential to further enhance PAR2 signaling as TF represents an important a co-factor for factor VII induced PAR2 activation (Camerer et al., 2000).

Activation of PAR2 is intimately related to the coagulation cascade, and mice lacking the *Par2/F2rl1* gene show reduced fibrin deposition in bleomycin-induced lung injury and glomerular nephritis (Moussa et al., 2007; Borensztajn et al., 2010; Bardou et al., 2016). Matriptase, a potent and highly specific PAR2-activating cell membrane-bound protease, has also been identified recently as a central coordinator of epithelial PAR2 activation, downstream of coagulation-related proteases TF-FVIIa and FXa (Le Gall et al., 2016). The specific roles for matriptase-PAR2 signaling in cellular mechanisms of physiology and disease, especially in the regulation of kidney hemostasis, remain unknown. As both trypsinogen and matriptase are expressed by kidney tubule cells, PAR2 may provide a molecular link between coagulation, epithelial proteases and kidney function (Koshikawa et al., 1998; Oberst et al., 2003).

In conclusion, this study shows that PAR2-activating proteases can activate HTEC to induce TF synthesis, secretion and blood clotting that is attenuated by PAR2 antagonists or a TF antibody. Our findings reveal a mechanism by which underlying hemostatic imbalances might increase the risk of thrombosis in the kidneys of human patients with CKD, and suggest PAR2 antagonism as a potential new therapeutic strategy for intervening in CKD progression.

## Data Availability Statement

The raw data supporting the conclusions of this article will be made available by the authors, without undue reservation.

## Ethics Statement

The studies involving human participants were reviewed and approved by Princess Alexandra Hospital Research Ethics Committee, Brisbane, Australia (ethics number: HREC/12/QPAH/125). The patients/participants provided their written informed consent to participate in this study.

## Author Contributions

DV and AI developed the concept, designed the experiments, performed the experimental work, analyzed the data, and wrote the manuscript. TH, PM, EO, and K-NZ performed the experimental work and analyzed the data. GG, DF, and DJ helped to write and edit the manuscript. DF provided specialized reagents. All authors contributed to the article and approved the submitted version.

## Conflict of Interest

DJ has received consultancy fees, research grants, speaker’s honoraria and travel sponsorships from Baxter Healthcare and Fresenius Medical Care, consultancy fees from Astra Zeneca and AWAK, speaker’s honoraria and travel sponsorships from ONO, and travel sponsorships from Amgen. DJ is a current recipient of an Australian National Health and Medical Research Council Practitioner Fellowship. DF is an inventor on a patent (AU20109033378) covering PAR2 agonists and antagonists that is owned by The University of Queensland. The remaining authors declare that the research was conducted in the absence of any commercial or financial relationships that could be construed as a potential conflict of interest.
