# Supplementary material for: PAR2 Activation on Human Kidney Tubular Epithelial Cells Induces Tissue Factor Synthesis, That Enhances Blood Clotting
Source: Front Physiol. 2021 Mar 10;12:615428. doi: 10.3389/fphys.2021.615428 (PMC7987918; doi:10.3389/fphys.2021.615428)
Supplement: Supplementary file 1 [file Table_1.DOCX]

**Supplementary Figure S1**

**Supplementary Figure 1**. **PAR2 activation induces a time dependent synthesis of Tissue Factor mRNA (A, n=3) and protein (B, C) by Human Tubular Epithelial cells in culture**. 1B is a representative western blot and C is a semi-quantitative representation of band densities for this blot. In A *p<0.05 compared to control treated cells. A Student’s T test was used.
